# Specialty Preferences and Intentions to Work in High-Need Contexts Among Graduating Sexual and Gender Minority Medical Students

**DOI:** 10.7759/cureus.103153

**Published:** 2026-02-07

**Authors:** Thomas M Freitag, Jeongyoung Park, Alison Huffstetler

**Affiliations:** 1 Psychiatry and Behavioral Sciences, Johns Hopkins University School of Medicine, Baltimore, USA; 2 Research, The Robert Graham Center for Policy Studies in Family Medicine and Primary Care, Washington, D.C., USA; 3 Medicine, The Robert Graham Center for Policy Studies in Family Medicine and Primary Care, Washington, D.C., USA; 4 Family Medicine and Population Health, Virginia Commonwealth University School of Medicine, Richmond, USA; 5 Family Medicine, Georgetown University School of Medicine, Washington, D.C., USA

**Keywords:** gender minority, lgbt health, medical education, medical education research, medically-underserved areas, primary care education, sexual and gender minorities, sexual minority, surgical-education, underserved populations

## Abstract

Introduction: Sexual minority (SM) and gender minority (GM) students make up a growing portion of medical student bodies and face unique challenges that shape their career decisions. Identifying what specialties draw interest from SM and GM medical students is crucial for anticipating the composition of an increasingly diverse clinical workforce and understanding what contribution these students could make to addressing health disparities faced by LGBTQIA+ patient populations.

Aim and objective: To study specialty choices and intent to work in underserved contexts among graduating SM and GM medical students in the United States and determine whether these differed from their heterosexual and cisgender counterparts.

Methods: We completed a secondary analysis of data from the 2022-2024 American Association of Medical Colleges’ Graduation Questionnaire (GQ), which included responses from 50,185 graduating allopathic medical school students. Respondents were stratified by gender identity, as well as sexual orientation and sex. Descriptive statistics were calculated to examine the specialty choices of SM and GM students. χ^2^ tests were used to compare intention to pursue primary care or surgical specialties and intention to work in underserved areas or with underserved populations.

Results: GM and SM medical students were more likely to intend to work in underserved areas (GM: n = 241/473, 50.95% vs. cisgender: n = 13,378/46,413, 28.82%; p < 0.001; SM: n = 2,205/5,713, 38.60% vs. heterosexual: n = 11,308/40,796, 27.72%, p < 0.001) and with underserved populations (GM: n = 326/473, 68.92% vs. cisgender: n = 18,454/46,409, 39.76%, p < 0.001; SM: n = 3,070/5,719, 53.68% vs. heterosexual: n = 15,563/40,787, 38.16%, p < 0.001) than their cisgender and heterosexual peers. GM and SM students were also less likely to pursue surgical specialties than their cisgender and heterosexual peers (GM: n = 96/469, 20.47% vs. cisgender: n = 12,120/46,117, 26.28%. p < 0.01; SM: n = 1,312/5,660, 23.18% vs. heterosexual: n = 10,812/40,553, 26.66%, p < 0.001). Family medicine (n = 93, 19.70%) and psychiatry (n = 69, 14.62%) were the most popular preferred specialties among graduating GM students, while internal medicine (n = 848, 14.85%) and psychiatry (n = 685, 11.99%) were most popular among SM students.

Conclusion: These results illustrate how GM and SM medical students are drawn toward specialties such as family medicine and psychiatry, which may be informed by these fields' perception as inclusive spaces. In addition, our findings demonstrate a decreased interest in surgical fields among GM and SM medical students. GM and SM students showed a greater propensity for intending to work in underserved clinical contexts and may become a core part of the clinical workforce supporting communities in need, such as LGBTQIA+ patients.

## Introduction

Sexual and gender minorities (SGMs) are defined as those with marginalized sexual orientations (SOs), gender identities (GIs), and/or reproductive development [1]. SGMs are estimated to make up over 9% of the adult U.S. population [2]. SGM patients have unique health needs that often go unmet and experience disparities in crucial primary care metrics and mental health outcomes [3,4]. SGM patients are less likely to seek out medical care and often face discrimination when receiving treatment [5-7]. Physicians have limited formal education on the health needs of SGM populations, which perpetuates these disparities [8,9]. Those who identify as SGMs make up a growing portion of medical student bodies, as younger generations with higher levels of LGBTQIA+ identification begin their undergraduate medical education [2,10].

Having providers with concordant identities has been found to foster improved health outcomes for marginalized populations, which suggests that developing a large workforce of SGM physicians could help advance SGM health [11,12]. However, there has been little research on the career decisions of SGM medical students. There is sparse literature on the career decisions of sexual minority (SM) medical students, whose SO differs from heterosexual norms, and even less on those of gender minority (GM) medical students, whose GI differs from their sex assigned at birth [13]. Determining what medical fields SGM students choose, or don’t choose, to pursue is important for understanding the factors that shape the experiences of SGM medical students as well as how the needs of SGM patients may be met (or unmet) in the future.

SGM medical students face challenges throughout the course of their education that may inform their specialty decisions. Research indicates that SGM students are more likely to face mistreatment during their undergraduate medical education and are more likely to view their learning environment negatively [14,15]. Some medical specialties are perceived to be less accepting of LGBTQIA+ people, which may lead SGM medical students to look elsewhere when making career decisions [16]. In contrast, research has found that SGM medical students perceive select specialties as being especially inclusive [17].

Furthermore, the forces that shape medical specialty decisions may differ between SGM and non-SGM medical students. For example, prior work has found the percentage of SGM trainees and physicians to be inversely related to specialty prestige and that SGM students were more likely to indicate that their SO and GI strongly influenced their specialty choice [17]. Others established that SM medical students were less likely to report that factors such as salary, residency length, and competitiveness influenced their choice of specialty when compared to their heterosexual peers [18].

A 2021 study by Mori et al. using Association of American Medical Colleges (AAMC) data found that SM medical students had a greater interest in pursuing specialties such as psychiatry and pathology, as well as differences in SM students’ likelihood of pursuing primary care or surgical specialties [19]. However, they were limited by a lack of GI data at the time of the original study, as the AAMC did not introduce comprehensive GI questions until 2022 [20]. The AAMC expanded response options for SO in 2022 as well [20].

We used data from the 2022-2024 AAMC Graduation Questionnaire (GQ) to characterize the medical specialty decisions of SGM medical students as well as their likelihood of intending to pursue primary care or surgical careers. We hypothesized that GM and SM medical students would gravitate toward specialties like family medicine, internal medicine, and psychiatry. We also expected that GM and SM medical students would be more likely to pursue primary care specialties and have a lower propensity for surgical specialties compared to their respective cisgender and heterosexual counterparts. We additionally hypothesized that GM and SM students would be more likely to work in high-need clinical contexts, such as underserved areas and with underserved populations.

## Materials and methods

We utilized deidentified self-reported data collected from 2022 to 2024 by the GQ organized by the AAMC. The GQ is a comprehensive survey sent to graduating MD students across the United States and includes questions about respondents’ medical specialty decisions and their intent to serve underserved areas or populations [20-22]. The AAMC obtained informed consent when conducting the original surveys. Additionally, we obtained data on respondents’ sex from previous AAMC data resources, primarily the AAMC account registration used for services such as the American Medical College Application Service. We performed a secondary analysis of GQ data using standard statistical methods. No proprietary scales or scoring systems were used for this project.

GM students were defined as those who selected something other than Man or Woman in response to the question, “What best describes your current gender identity?” This included those who selected Trans Man, Trans Woman, Agender, Genderqueer/Gender non-conforming, Non-binary, or Other/Another Gender Identity (free response). SM students were defined as those who selected something other than Heterosexual or Straight when responding to the question, “What best describes your current sexual orientation?”, including those who selected Asexual, Bisexual, Gay and Lesbian, Pansexual, Queer, and Other/Another Sexual Orientation (free response). Respondents without GI or SO information were excluded from their respective stratified analyses.

Intended specialty was defined utilizing criteria originally outlined by Mori et al. in 2021 and was based on responses to the question, “When thinking about your career, what is your intended area of practice?” Primary care specialties were defined as Family Practice or subspecialty, Internal Medicine, Pediatrics, and Internal Medicine/Pediatrics. Surgical specialties were defined as Neurological Surgery, Obstetrics and Gynecology, Ophthalmology, Orthopaedic Surgery or subspecialty, Otolaryngology or subspecialty, Plastic Surgery, General Surgery, Vascular Surgery, Thoracic Surgery, and Urology [19]. Survey entries without intended specialty information were excluded from analyses on intended specialty as well as intent to pursue primary care or surgical specialties. Specialty choices with fewer than 100 total responses from all survey respondents were excluded to prevent distortions caused by small sample sizes.

The intent to work in an underserved area was defined using responses to a question that asked, “Do you plan to work primarily in an underserved area?” Respondents could select Yes, No, or Undecided. The intent to work with underserved populations was defined using responses to a question that asked, “Regardless of location, do you plan to care primarily for an underserved population?” Respondents could select Yes, No, or Undecided. Survey entries without the answer to these questions were excluded from analyses on intent to work in underserved areas or with underserved populations.

Summary statistics on SM and GM specialty preferences, as well as SM and GM representation by intended specialty, were calculated. Specialty choices and intent to work in underserved areas/with underserved populations were compared using χ^2^ tests, stratified by GI and SO. GM respondents were compared with cisgender respondents, and SM respondents were compared with heterosexual respondents. We further stratified by sex for the SO analysis. All statistical analyses were performed using Stata version 19 (StataCorp LLC, College Station, TX, USA). Figures were prepared in GraphPad Prism version 10.5 (GraphPad Software, Boston, MA, USA).

## Results

Descriptive statistics

There were a total of 50,185 distinct records included across the three survey years, with response rates of 80.3%, 79.8%, and 79.5% in 2022, 2023, and 2024, respectively [20-22]. Of these responses, 3,098 (6.17%) did not have information on GI. Fifty-six (0.11%) students selected other/another GI but left their free response portion blank, included a non-response, or submitted non-viable data. These 3,154 responses were removed from analyses stratified by GI. A total of 47,031 respondents were included for the GI analysis (Table 1). A total of 3,402 (6.78%) entries did not have information on SO, and 148 (0.29%) respondents selected other/another SO but left their free response portion blank, gave a non-response, or submitted non-viable data. These 3,550 responses were removed from analyses stratified by SO. A total of 46,635 respondents were included for the SO analysis (Table 1). Eight respondents did not have sex data and were excluded from analyses stratified by SO and sex.

**Table 1:** Descriptive Statistics

Gender Minority Analysis	
Respondent Gender Identity (n = 47,031)	Frequency (Percentage)
Man	21,167 (45.01)
Woman	25,390 (53.99)
Trans Man	41 (0.09)
Trans Woman	14 (0.03)
Agender	36 (0.08)
Genderqueer or Gender Non-conforming	146 (0.31)
Non-binary	221 (0.47)
Other/Another Gender Identity	16 (0.03)
Gender Minority Respondents (total)	474 (1.01)
Sexual Minority Analysis	
Respondent Sexual Orientation (n = 46,635)	Frequency (Percentage)
Heterosexual or Straight	40,911 (87.73)
Asexual	284 (0.61)
Bisexual	2,581 (5.53)
Gay or Lesbian	1,869 (4.01)
Pansexual	281 (0.6)
Queer	664 (1.42)
Other/Another Sexual Orientation	45 (0.1)
Sexual Minority Respondents (total)	5,724 (12.27)
Sexual Minority Male Analysis	
Respondent Sexual Orientation (n = 21,168)	Frequency (Percentage)
Heterosexual or Straight	18955 (89.55)
Asexual	68 (0.32)
Bisexual	524 (2.48)
Gay or Lesbian	1396 (6.59)
Pansexual	61 (0.29)
Queer	148 (0.70)
Other/Another Sexual Orientation	16 (0.08)
Male Sexual Minority Respondents (total)	2213 (10.45)
Sexual Minority Female Analysis	
Respondent Sexual Orientation (n = 25,459)	Frequency (Percentage)
Heterosexual or Straight	21954 (86.23)
Asexual	216 (0.85)
Bisexual	2055 (8.07)
Gay or Lesbian	473 (1.86)
Pansexual	220 (0.86)
Queer	512 (2.01)
Other/Another Sexual Orientation	29 (0.11)
Female Sexual Minority Respondents (total)	3,505 (13.77)

Of the 47,031 respondents included in the GI analysis, 474 were categorized as GM (1.01% (95% CI 0.92%-1.10%)). A total of 21,167 were identified as men, 25,390 as women, 41 as transgender men, 14 as transgender women, 36 as agender, 146 as genderqueer or gender non-conforming, 221 as non-binary, and 16 as other/another GI (Table 1). Of the 46,635 respondents included in the SO analysis, 5,724 were categorized as SM (12.27% (95% CI 11.98%-12.56%)). A total of 40,911 identified as heterosexual, 284 as asexual, 2,581 as bisexual, 1,869 as gay or lesbian, 281 as pansexual, 664 as queer, and 45 as other/another SO (Table 1). Of the 46,627 respondents included in analyses stratified by SO and sex, 25,459 were female, and 21,168 were male (Table 1). A total of 3,505 female respondents identified as SM (13.77% (95% CI 13.34%-14.18%)), and 2,213 male respondents identified as SM (10.45% (95% CI 10.04%-10.86%)). SM students were more likely to be female (61.30% (3,505 of 5,718) of SMs vs. 53.67% (21,954 of 40,909) heterosexuals; p < 0.001).

Specialty choices by GI, SO, and SO + sex

**Figure 1:**
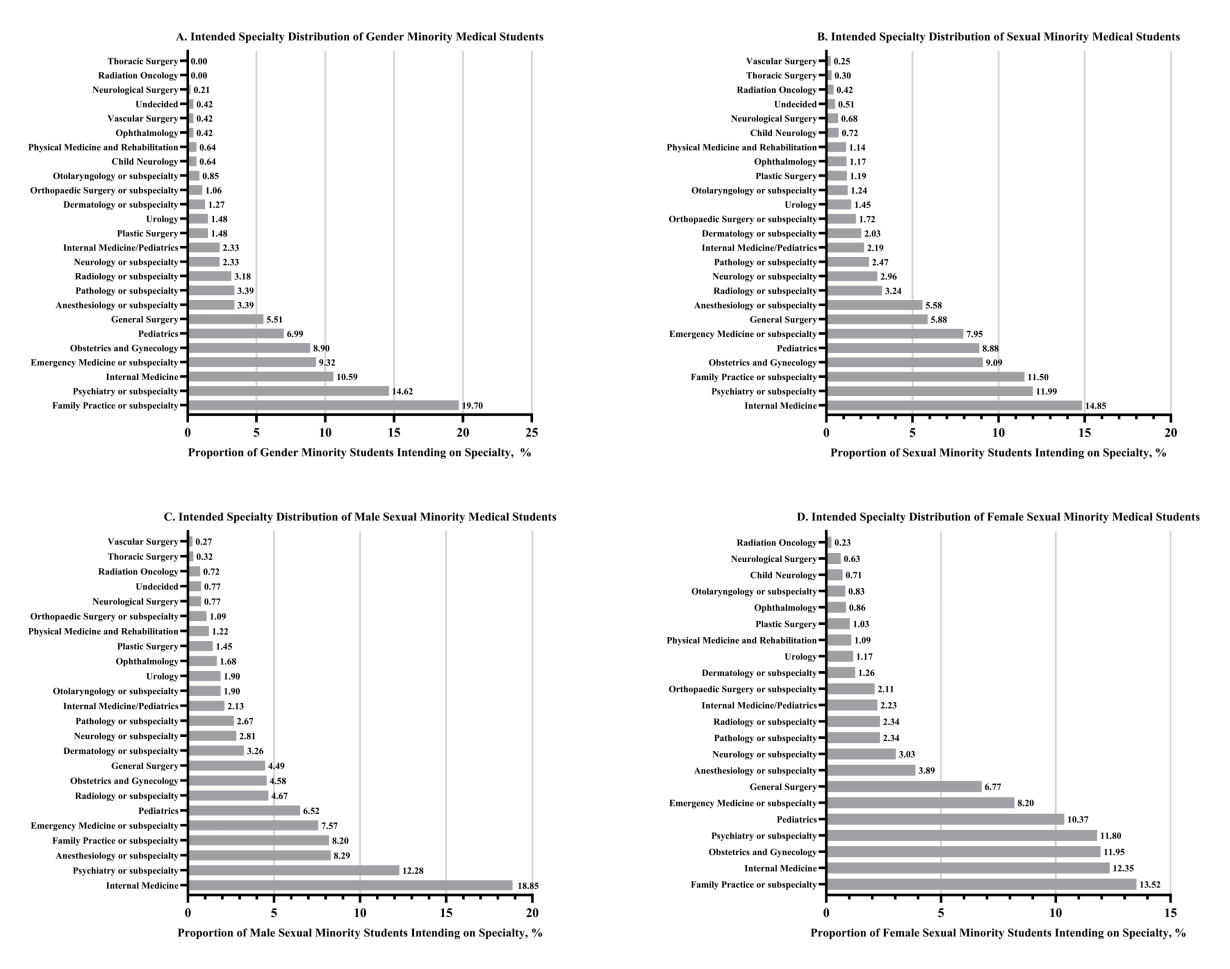
Specialty Preferences of Graduating Medical Students by Gender Identity, Sexual Orientation, and Sexual Orientation + Sex

Family practice or subspecialty (n = 93, 19.70%) and psychiatry or subspecialty (n = 69, 14.62%) were the most popular intended specialties among GM students (Figure 1). Zero GM students selected thoracic surgery (246 total responses) or radiation oncology (253 total responses) as their intended specialty (Figure 1). Internal medicine (n = 848, 14.85%) and psychiatry or subspecialty (n = 685, 11.99%) were the most popular intended specialties among SM students, while vascular surgery (n = 14, 0.25%) was the least popular (Figure 1). When stratified by sex, internal medicine (n = 416, 18.85%) and psychiatry or subspecialty (n = 271, 12.28%) were the most popular intended specialties among male SM students, whereas family practice or subspecialty (n = 473, 13.52%) and internal medicine (n = 432, 12.35%) were the most popular among female SM students (Figure 1).

**Figure 2:**
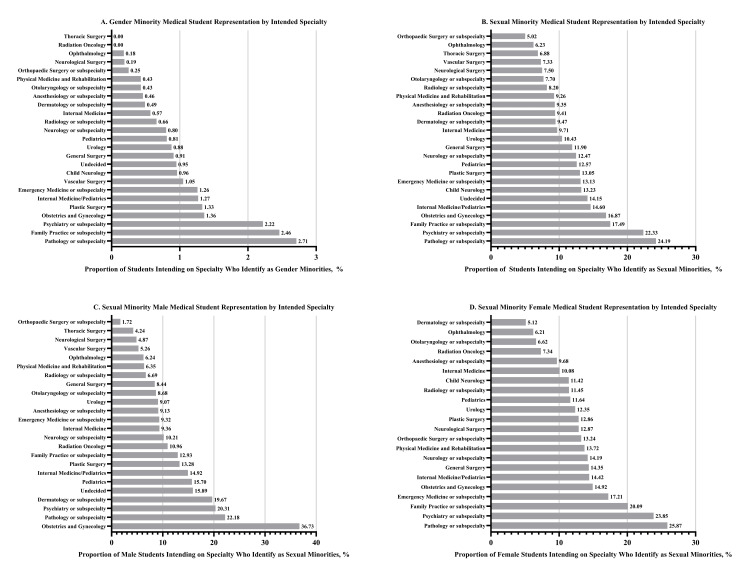
Sexual and Gender Minority Medical Student Representation by Intended Specialty

Pathology or subspecialty (n = 16/590, 2.71%) and family practice or subspecialty (n = 93/3788, 2.46%) had the highest representation of GM students among those intending to match in the field (Figure 2). Pathology or subspecialty (n = 141/583, 24.19%) and psychiatry or subspecialty (n = 685/3,068, 22.33%) had the highest SM representation, whereas orthopedic surgery (n = 98/1,951, 5.02%) had the lowest (Figure 2). Obstetrics and gynecology (n = 101/275, 36.73%) had the highest representation of SM males, and pathology or subspecialty (n = 82/317, 25.87%) had the highest representation of SM females (Figure 2). Orthopedic surgery (n = 24/1,392, 1.72%) and dermatology or subspecialty (n = 44/859, 5.12%) had the lowest representation of SM males and SM females, respectively (Figure 2).

Intention to pursue primary care or surgical specialties

Compared to their cisgender peers, GM students were significantly less likely to intend to pursue surgical specialties (n = 96/469, 20.47% vs. n = 12,120/46,117, 26.28%; p < 0.01) (Table 2). SM students were significantly less likely to intend to pursue surgical specialties than their heterosexual counterparts (n = 1,312/5,660, 23.18% vs. n = 10,812/40,553, 26.66%; p < 0.001) (Table 2).

**Table 2:** Intention to Pursue Primary Care and Surgical Specialties by Gender Identity, Sexual Orientation, and Sexual Orientation + Sex

Category	Group	Intention to Pursue Primary Care Specialty			Intention to Pursue Surgical Specialty		
		% Pursuing Primary Care Specialty	Pearson's Chi^2^	p-value	% Pursuing Surgical Specialty	Pearson's Chi^2^	p-value
Gender Identity	Gender Minority	187/469 (39.87%)	1.07	0.30	96/469 (20.47%)	8.11	<0.01
	Cisgender	17,316/46,117 (37.55%)			12,120/46,117 (26.28%)		
Sexual Orientation	Sexual Minority	2,137/5,660 (37.76%)	0.07	0.80	1,312/5,660 (23.18%)	31.1	<0.001
	Heterosexual	15,240/40,553 (37.58%)			10,812/40,553 (26.66%)		
Sexual Orientation (Male)	Sexual Minority Male	788/2,174 (36.25%)	6.53	0.01	407/2,174 (18.72%)	51.97	<0.001
	Heterosexual Male	6,289/18,768 (33.51%)			4,842/18,768 (25.80%)		
Sexual Orientation (Female)	Sexual Minority Female	1,346/3,480 (38.68%)	7.24	<0.01	905/3,480 (26.01%)	2.95	0.09
	Heterosexual Female	8,951/21,783 (41.09%)			5,969/21,783 (27.40%)		

SM male students were significantly more likely to intend to pursue primary care specialties (n = 788/2,174, 36.25% vs. n = 6,289/18,768, 33.51%; p = 0.01) and significantly less likely to intend to pursue surgical specialties (n = 407/2,174, 18.72% vs. n = 4,842/18,768, 25.80%; p < 0.001) than heterosexual males (Table 2). SM female students were significantly less likely to intend to pursue primary care specialties (n = 1,346/3,480, 38.68% vs. n = 8,951/21,783, 41.09%; p < 0.01) when compared with their heterosexual female peers (Table 2).

Intention to work in underserved areas and with underserved populations

GM medical students were significantly more likely to intend to work in underserved areas (n = 241/473, 50.95% vs. n = 13,378/46,413, 28.82%; p < 0.001) and with underserved populations (n = 326/473, 68.92% vs. n = 18,454/46,409, 39.76%; p < 0.001) than cisgender students (Table 3). SM medical students were significantly more likely to intend to work in underserved areas (n = 2,205/5,713, 38.60% vs. n = 11,308/40,796, 27.72%; p < 0.001) and with underserved populations (n = 3,070/5,719, 53.68% vs. n = 15,563/40,787, 38.16%; p < 0.001) than heterosexual students (Table 3).

**Table 3:** Intention to Work in Underserved Contexts by Gender Identity, Sexual Orientation, and Sexual Orientation + Sex

Category	Group	Intention to Work in an Underserved Area			Intention to Work With Underserved Populations		
		% Intending to Work in an Underserved Area	Pearson's Chi^2^	p-value	% Intending to Work With Underserved Populations	Pearson's Chi^2^	p-value
Gender Identity	Gender Minority	241/473 (50.95%)	111.24	<0.001	326/473 (68.92%)	165.79	<0.001
	Cisgender	13,378/46,413 (28.82%)			18,454/46,409 (39.76%)		
Sexual Orientation	Sexual Minority	2,205/5,713 (38.60%)	287.66	<0.001	3,070/5,719 (53.68%)	503.37	<0.001
	Heterosexual	11,308/40,796 (27.72%)			15,563/40,787 (38.16%)		
Sexual Orientation (Male)	Sexual Minority Male	718/2,211 (32.47%)	97.11	<0.001	989/2,209 (44.77%)	206.93	<0.001
	Heterosexual Male	4,351/18,906 (23.01%)			5,626/18,900 (29.77%)		
Sexual Orientation (Female)	Sexual Minority Female	1,484/3,496 (42.45%)	154.45	<0.001	2,076/3,504 (59.25%)	232.12	<0.001
	Heterosexual Female	6,957/21,888 (31.78%)			9,937/21,885 (45.41%)		

SM males were significantly more likely to intend to work in underserved areas (n = 718/2,211, 32.47% vs. n = 4,351/18,906, 23.01%; p < 0.001) and with underserved populations (n = 989/2,209, 44.77% vs. n = 5,626/18,900, 29.77%; p < 0.001) than heterosexual males (Table 3). SM female students were significantly more likely to intend to work in underserved areas (n = 1,484/3,496, 42.45% vs. n = 6,957/21,888, 31.78%; p < 0.001) and with underserved populations (n = 2,076/3,504, 59.25% vs. n = 9,937/21,885, 45.41%; p < 0.001) when compared to heterosexual females (Table 3).

## Discussion

Recent cohorts of graduating SGM medical students have distinct preferences for specialties such as family medicine and psychiatry, suggestive of both inclusivity among these specialties as well as bias and exclusion in other fields. In addition, SGM students demonstrated a propensity for working in high-need clinical contexts, being significantly more likely to express interest in practicing in underserved areas and with underserved populations. This study is the first to characterize the intended specialty choices of graduating GM medical students and builds on the work of Mori et al. in describing the career intentions of SGM medical students.

Our findings regarding the specialty choices of SGM students may be explained by a number of factors. The tendency for SGM students to be drawn toward fields like family medicine and psychiatry may reflect a perception that these specialties have cultures and patient populations that are accepting of LGBTQIA+ providers. Prior research has demonstrated that SGM medical students see specialties like psychiatry and family medicine as being especially inclusive [17]. In addition, the tendency of these fields to care for SGM populations and address SGM-specific clinical concerns (e.g., HIV, mental health disparities) could lead SGM students to feel that they can address the needs of their communities. Although specialties like psychiatry have complex histories of stigmatizing SGM patients and physicians, their current iterations have championed the needs of LGBTQIA+ communities [23-25].

SGM medical students’ tendency to be less interested in surgical specialties may reflect a lack of perceived inclusivity and opportunities to address SGM health needs. Research has found that surgical specialties are seen as being less inviting to LGBTQIA+ medical students [16]. This idea of surgery as being hostile to SGM trainees is further propagated by higher rates of discrimination and harassment faced by SGM surgical residents [26]. Furthermore, surgery’s troubled history of strained relationships with LGBTIQA+ communities may lead SGM medical students to expect that they will have fewer opportunities to support patients who share their identity [27].

Graduating SGM medical students’ interest in working in underserved areas and with underserved populations could be informed by their experiences as members of marginalized communities, as well as a desire to serve populations that share their identity. Facing discrimination or mistreatment on the basis of SO/GI may instill a desire to rectify similar injustices. In addition, SGM students’ lower propensity to prioritize factors such as high compensation or prestige may create opportunities to pursue specialties that prioritize serving those in need [17,18].

When comparing our results to those of Mori et al., several findings stand out. First, the proportion of SM students in our sample was nearly twice that of the proportion of SM students in the original study (12.27% vs 6.3%, respectively) [19]. This likely reflects changes in the demographic composition of medical school cohorts graduating in 2022-2024 vs. 2016-2019. Additionally, expanded SO options offered starting in 2022 may have facilitated more disclosures, and SM students may have become more comfortable disclosing their SO in major surveys like the GQ [2,20].

In addition, our study found that approximately 1% of respondents from the 2022-2024 GQ identified as GM. Estimates on the percentage of young adults who identify as GM in the United States vary, ranging from about 0.5% to 2.7% depending on the specific measures used (such as whether those who identify as gender nonconforming or non-binary are included) [28]. GM identification has steadily increased among younger generations, with a significant rise among those born after 1997 [28]. Assuming that capture of GI in the GQ continues, we might expect that GM identification will become more prevalent in subsequent iterations of the survey. Finally, our finding that 12.27% of respondents identified as SM is notable, given that this could actually be lower than the proportion of adults who identify as SM in the general population (with some estimates of Millennial LGBTQIA+ identification as high as 14.2%) [2,29].

Limitations

Although the GQ is a rich source of data on the career decisions of graduating medical students, our study suffered from some notable limitations. The GQ only captures responses from students enrolled in allopathic medical schools, leaving us with no insights into the career decisions of SGM osteopathic medical students. This is particularly notable given the higher propensity of osteopathic medical school graduates to work in primary care [30]. The GQ also captured a small number of GM medical students, with less than 500 GM respondents from a pool of over 50,000 students across three years. Although the GQ has strong response rates of approximately 80% per year, results among non-respondents may differ. Additionally, some students may not have disclosed that they are GMs, meaning that responses could reflect a subset of “out” students whose specialty and career preferences may differ from those of GM medical students as a whole.

Finally, the GQ does not include information on graduating student grades, test scores, or technical data that could have allowed us to examine whether SGM students were as competitive residency applicants as their non-SGM peers. If SGM students were, on average, less competitive applicants, some of their intended specialty choices may reflect realistic considerations about matching into less competitive fields (such as family medicine) rather than their true personal preferences. As a result, we may incorrectly interpret these specialty choices as reflecting factors such as an interest in public service, when they may instead be influenced by structural barriers, including discrimination, that limit SGM students’ ability to develop competitive residency applications.

## Conclusions

This study explored the career decisions of SM and GM medical students, highlighting the importance of inclusivity in medical education and providing some of the first descriptions of the specialty interests of graduating GM medical students. SM and GM students demonstrated an interest in pursuing fields such as family practice and psychiatry, as well as a desire to work in underserved clinical contexts. Fields such as pathology, family practice, and psychiatry demonstrated high rates of SM and GM representation among graduating medical students who intended to pursue their fields. In addition, SM and GM medical students tended to be less likely to express an interest in pursuing surgical specialties, which could reflect a perceived lack of inclusivity within those spaces. These results are particularly important given the present sociopolitical hostility towards SGM people as well as the persistence of poorer health outcomes and difficulty accessing healthcare services among LGBTQIA+ communities. Our findings suggest that SM and GM medical students may contribute to a physician workforce that is drawn to fields and populations where there is a high need, such as LGBTQIA+ patients.
